# Diagnosis of Infections Caused by Pathogenic Free-Living Amoebae

**DOI:** 10.1155/2009/251406

**Published:** 2009-08-02

**Authors:** Bruno da Rocha-Azevedo, Herbert B. Tanowitz, Francine Marciano-Cabral

**Affiliations:** ^1^Department of Microbiology and Immunology, Virginia Commonwealth University School of Medicine, Richmond, VA 23298, USA; ^2^Department of Pathology, Albert Einstein College of Medicine, Bronx, NY 10461, USA

## Abstract

*Naegleria fowleri*, *Acanthamoeba spp.*, *Balamuthia mandrillaris*, and *Sappinia* sp. are pathogenic free-living amoebae. *N. fowleri* causes Primary Amoebic Meningoencephalitis, a rapidly fatal disease of the central nervous system, while *Acanthamoeba spp.* and *B. mandrillaris* cause chronic granulomatous encephalitis. *Acanthamoeba spp.* also can cause cutaneous lesions and Amoebic Keratitis, a sight-threatening infection of the cornea that is associated with contact lens use or corneal trauma. *Sappinia pedata* has been identified as the cause of a nonlethal case of amoebic encephalitis. In view of the potential health consequences due to infection with these amoebae, rapid diagnosis is critical for early treatment. Microscopic examination and culture of biopsy specimens, cerebral spinal fluid (CSF), and corneal scrapings have been used in the clinical laboratory. For amoebic keratitis, confocal microscopy has been used to successfully identify amoebae in corneal tissue. More recently, conventional and real-time PCR assays have been developed that are sensitive and specific for the amoebae. In addition, multiplex PCR assays are available for the rapid identification of these pathogens in biopsy tissue, CSF, and corneal specimens.

## 1. Introduction

**Figure 1:**
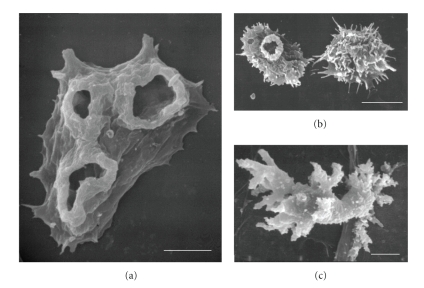
Fine morphology of trophozoites of *N. fowleri,* (a) *Acanthamoeba spp.*, (b) and *B. mandrillaris*, (c) by Scanning Electron Microscopy. Bars represent 5 *μ*m.

Free-living amoebae (FLA) are found in soil and water habitats throughout the world. These amoebae ingest bacteria, yeast, and other organisms as a food source. Unlike “true” parasites, pathogenic FLA can complete their life cycles in the environment without entering a human or animal host. Of the many FLA that are found in the environment, four genera of FLA have been associated with human disease. One species of *Naegleria*, *N. fowleri,* one species of *Balamuthia, B. mandrillaris*, and several species of *Acanthamoeba* can cause fatal central nervous system (CNS) infections. In addition to a CNS infection, *Acanthamoeba* can cause cutaneous lesions and Amoebic Keratitis, a sight-threatening infection of the cornea [1–8]. Human infections with these amoebae have been reported from all over the world [3, 5]. More recently, a newly recognized pathogen was detected in brain tissue of a patient with CNS symptoms who survived the infection. Although the amoebae was not isolated from the patient, it was identified by light and electron microscopy as a species of *Sappinia*. *Sappinia* sp. has not been shown to be lethal in humans or experimental animals [9, 10]. Figure 1 shows the morphology of trophozoites of *N. fowleri* (A), *Acanthamoeba spp.* (B), and *B. mandrillaris* (C), by scanning electron microscopy.

Pathogenic FLA can be isolated from freshwater lakes, thermally polluted waters, sediment, thermal springs, swimming pools, soil, air conditioning vents, air, and the domestic water supply [3, 6, 11–14]. In addition to causing human disease, FLA also can harbor intracellular pathogenic bacteria such as *Legionella pneumophila* and may serve as vectors of bacterial infections in humans [15, 16]. A number of clinical FLA isolates from corneal and cutaneous lesions have been shown to harbor bacterial endosymbionts or pathogens [16–20]. Laboratory studies, also, have shown that a number of pathogenic bacteria including *Mycobacterium avium, Burkholderia spp.*, *Escherichia coli O157:H7*, and *Vibrio cholerae*, can survive and multiply in FLA [6, 21–26]. Intracellular growth of bacteria within amebae has been shown to increase bacterial resistance to antibiotics and to biocides, and to increase bacterial virulence [20, 23–26]. 

Since the majority of FLA infections are fatal and diagnosed postmortem, it is important to recognize the diseases and to develop more rapid diagnostic methods. The mode and pathogenesis of infection differ for each of the FLA that cause human infections. 

## 2. *Naegleria fowleri * and Primary Amoebic Meningoencephalitis (PAM)

The amoeboflagellate, * N. fowleri,* has three morphological forms in its life cycle, an amoeba or trophozoite stage (Figure 1(a)) that feeds and divides, a swimming flagellate that seeks out a new food source, and a resistant cyst that protects the amoebae from adverse environmental conditions. *N. fowleri* causes Primary Amoebic Meningoencephalitis (PAM), an acute, fulminant, rapidly fatal disease that occurs generally in previously healthy children and young adults with a history of swimming and diving and other recreational activities in fresh water and contaminated swimming pools [1–3, 27–29]. Two other species of *Naegleria*, *N. australiensis* and *N. italica* can cause infections in mice but have never been identified from human infections [5]. Cases of PAM caused by *N. fowleri* have occurred also from contaminated domestic water used for bathing [11, 12]. Infection can occur when amebae enter the nasal passages, attach to the olfactory mucosa, and migrate through the cribriform plate alongside the olfactory nerves. Once in the olfactory bulbs of the brain, the amebae divide rapidly and death occurs within 7 to 10 days. *N. fowleri* causes a fulminating hemorrhagic necrosis of the brain. An inflammatory infiltrate consisting of neutrophils, eosinophils, and macrophages is histopathological characteristic of infected brain tissue. Only trophozoites are found in the brain [3–5, 29]. Survival from PAM is dependent on rapid diagnosis and treatment of the disease and has occurred when the disease was recognized early and treatment initiated promptly [30, 31]. Survival rate is improved when a combination of amphotericin B is used intravenously, with intrathecal administration of amphotericin B and oral rifampin and other antifungal agents [30–34]. Amphotericin B and fluconazole administered intravenously followed by oral rifampicin resulted in successful treatment of a child who developed PAM [35]. However, not all patients treated with Amphotericin B survive [36–38]. Others have suggested that a triple combination of low dose amphotericin B administered intravenously (IV) with oral rifampacin and oral ketoconazole would result in a more favorable outcome [39]. Azithromycin has been shown to be effective against *N. fowleri* in vitro (cell culture) and in vivo (mouse model of infection) [8]. However, optimal treatment remains to be developed. 

### 2.1. Symptoms of PAM Infection

PAM is characterized by the sudden onset of severe frontal headache, fever, nausea, vomiting, and rhinitis. These are followed by stiff neck, diplopia, loss of the sense of smell, confusion, and occasional seizures, progressing rapidly to coma and death. An elevated white cell count is usual with a marked increase in neutrophils. CSF contains neutrophils, and thus a bacterial infection is often suspected [3–5, 7]. A history that describes contact with warm water (diving, wakeboarding, water skiing) is suggestive of PAM [40]. 

### 2.2. Laboratory Diagnosis

PAM is a rare disease but almost always fatal. Therefore, early diagnosis is important in order to start treatment. The disease is often misdiagnosed because no distinctive differences in diagnosis exist to distinguish PAM from bacterial meningoencephalitis.

#### 2.2.1. Imaging Methods

Computed Tomography (CT) scans or Magnetic Resonance Imaging (MRI) shows lesions but these are nonspecific [41–43]. CT scans show obliteration of the cisterns surrounding the midbrain and the subarachnoid space [3].

#### 2.2.2. Microscopic Methods

Premortem diagnosis is rare, but when CSF pressure is low, lumbar puncture can be performed. CSF is purulent, and when bacteria are not found, amoebic meningoencephalitis should be considered. The CSF is cloudy and slightly hemorrhagic with increased cellularity composed mainly of neutrophils. CSF is characterized by low glucose and elevated protein. Direct microscopic examination of CSF as a wet mount is the method of choice in the diagnosis of PAM because CSF contains motile amoebae which can be recognized by light microscopic observation [5, 44, 45]. *N. fowleri* can be distinguished from other FLA that cause CNS infections because amoebae can transform into swimming flagellates when amoebae are placed in water. If present in CSF, amoebae can be identified by staining fixed preparations with Wright's, Giemsa, or hematoxylin and eosin (H & E). Although Gram stain is used in clinical laboratories for detection of bacteria in CSF, Gram stain is not useful for diagnosis of amoebae because it does not depict the characteristic nuclear morphology of the amoebae. Amoebae can be mistaken as macrophages, but *N. fowleri* nucleus contains a large, central, round nucleolus which should distinguish it from host cells [3, 27, 44, 45]. 

Polyclonal antibodies produced in rabbits or monoclonal antibodies can be used to identify amoebae in tissue sections and CSF. Amoebae in CSF can be identified by specific indirect immunofluorescent antibody assays using a polyclonal or monoclonal antibody to the amoeba in conjunction with a flouresceinated secondary antibody [3, 45, 46]. 

Biopsy material should be fixed in 10% neutral buffered formalin for histological examination. Amoebae can be observed in biopsied brain tissue following H & E staining or by immunoperoxidase staining using antibodies to the amoebae. Only trophozoites are found in brain tissue; cysts are not observed [3]. 

A commercially available enzyme-linked immunosorbent assay (Indicia, Oulin, France) based on the use of a monoclonal antibody (5D12) that recognizes a glycosylated epitope on *N. fowleri* can be used to diagnose infections. This monoclonal antibody can be used to distinguish *N. fowleri* from other species of *Naegleria*, and from other FLA in tissue and in environmental samples [46, 47].

#### 2.2.3. Culture Methods

CSF or biopsied brain tissue should be kept and transported at room temperature to the diagnostic laboratory. This material can be inoculated onto tissue culture cells (Vero, fibroblasts) and incubated at 37°C in the presence of the antibiotics, penicillin-streptomycin. Fungicides are lethal to the amoebae. Amoebae that are present will multiply and destroy the monolayer in 24 to 48 hours. Biopsy tissue also can be placed on 1.5% nonnutrient agar coated with a layer of bacteria (*Escherichia coli*). The amoebae will emerge from the tissue, ingest the bacteria, and divide. The amoebae, then, can be observed on the agar using an inverted light microscope [3, 44, 45, 48]. 

#### 2.2.4. Serology

Antibodies to *Naegleria spp*. have been identified in healthy individuals [49, 50]. Since PAM is a rapid disease, serological tests for an increase in antibody titer are not always helpful. Generally, there is not a rise in antibody titer although a rise in antibody has been observed in a patient that was successfully treated and survived the infection [30].

#### 2.2.5. Polymerase Chain Reaction (PCR) Assays

More rapid molecular techniques are now available in research laboratories, but these methods generally are not available in most clinical laboratories. Highly specific and sensitive PCR and real-time PCR assays have been developed for the detection of *N. fowleri* in clinical and environmental samples [51–57]. A PCR assay using primers for the complete ribosomal internal transcribed spacer region (ITS) has been developed that allows for the discrimination of *Naegleria* species, and a species specific assay allows for detection of *N. fowleri* [51, 52]. A PCR assay that detects *N. fowleri* in fresh brain tissue as well as in formalin-fixed paraffin-embedded brain tissue also has been reported [56]. Recently, Qvarnstrom et al., [57] developed a fast and sensitive multiplex real-time PCR assay based on the use of probes targeting the partial or full length nuclear small subunit ribosomal genes (18S rRNA gene) for simultaneous detection of *Naegleria*, *Balamuthia,* and *Acanthamoeba* [57]. This PCR assay is species specific for *N. fowleri* and *B. mandrillaris* and genus specific for *Acanthamoeba.* Thus, this assay can identify which amoebae is present in a CSF sample or a brain biopsy specimen from an amoebic encephalitis patient. The detection limit for this assay was shown to be one amoebae per sample. 

## 3. *Acanthamoeba spp.*

*Acanthamoeba* is one of the most commonly isolated amoebae in environmental samples. *Acanthamoeba* is ubiquitous and found in a variety of habitats including domestic water supplies, hospital water, dental water units, air, soil, and water. *Acanthamoeba* has two morphological forms in its life cycle, a trophozoite (Figure 1(b)) and a cyst stage. Both stages can be found in tissues of infected humans and in the environment. The trophozoite is the dividing form and is thought to be the infective stage. The cysts are dormant and protect the amoebae from harmful environments. The cysts are resistant to biocides, chlorination, and antibiotics. Several species of *Acanthamoeba* can cause Granulomatous Amoebic Encephalitis (GAE), cutaneous acanthamoebiasis, or Amoebic Keratitis (AK). AK is a sight-threatening infection of the cornea that occurs in immune competent individuals, mainly contact lens users. GAE, also known as *Acanthamoeba* Granulomatous Encephalitis (AGE), is a rare, chronic, progressive infection of the CNS that may involve the lungs [58]. GAE is usually associated with an underlying debilitating disease or immune suppressed individuals including HIV-AIDS patients, diabetics, individuals undergoing organ transplants or cancer chemotherapy, and drug abusers [6, 7, 58, 59]. Cutaneous lesions caused by *Acanthamoeba* also have been described. Cutaneous acanthamoebiasis has been reported more frequently in HIV positive patients than in other conditions [60–66]. The manifestations of cutaneous infection include the presence of numerous hard erythematous nodules, papules, or ulcers along the patient's body [62–67]. The presence of both skin lesions and CNS symptoms occurring simultaneously can be suggestive of an *Acanthamoeba* infection [58]. 

### 3.1. Symptoms of GAE

GAE symptoms include headaches, slight fever, seizures, hemiparesis, cranial nerve palsies, personality changes, nausea, stiff neck, depressed level of consciousness and coma, typical clinical signs of a localized encephalopathy [5–8]. The clinical signs of GAE are not specific. Thus, the disease is often misdiagnosed as bacterial leptomeningitis, tuberculous meningitis, viral encephalitis, toxoplasmosis, fungal infections, neurocysticercosis, or a brain tumor [5, 6, 68–71]. 

### 3.2. Diagnostic Methods

#### 3.2.1. Imaging Methods

Brain imaging methods, such as CT and MRI, have been used to visualize brain lesions caused by *Acanthamoeba*, but the lesions themselves are not specific for GAE [41–43]. Multifocal low-density lesions in both cortical and subcortical regions of the brain can be observed using CT scans. Enhanced CT normally shows the presence of progressive hydrocephalus, with meningeal thickening, pseudotumoral lesions, large isolated lesions, or multiple oval lesions. Multifocal lesions, edema, and multiple ring-enhancing lesions are commonly observed in GAE patients by MRI. Despite these characteristics, both CT and MRI have limited diagnostic value for GAE [5, 7, 41–43, 68–73].

#### 3.2.2. Microscopic Methods

The definitive diagnosis of GAE is the detection of the amoeba in tissue or isolation of the amoeba. To achieve visual detection of both *Acanthamoeba* trophozoites and cysts in brain tissue, skin lesions, or cerebrospinal fluid (CSF), both light and electron microscopy can be used. *Acanthamoeba* trophozoites can be distinguished from host inflammatory cells such as macrophages mainly by their nuclear structure, since *Acanthamoeba* possesses a rounded nucleus and a large central nucleolus, forming a halo [3, 6, 59]. However, it is not possible to differentiate *Acanthamoeba* trophozoites from pathogenic *B. mandrillaris* trophozoites by light microscopy, since both amoebae possess the same nuclear structure [7]. Biopsy or autopsy specimens should be formalin-fixed, paraffin-embedded, and stained with H & E [74]. Other types of histological staining have been used, including Periodic Acid Shiff, Gomori's methenamine silver, or trichome. These stains appear to be effective in identifying cysts [75, 76]. Acridine orange and calcofluor white have been used successfully to observe *Acanthamoeba* cysts in tissues [6, 77, 78]. 

Brain granulomas, necrosis with the presence of multinucleated cells, inflammatory infiltrates, and amebas (both trophozoites and cysts) surrounding blood vessels [3–5] can be observed in biopsied tissue stained with H & E. Amoebae, also, can be detected in CSF in wet preparations or Giemsa stained slides of CSF sediments [29, 79, 80]. For the diagnosis of cutaneous acanthamoebiasis, light microscopic examination of H & E stained skin biopsies demonstrates the presence of granulomas, areas of necrosis, inflammatory infiltrates, and vasculitis containing both trophozoites and cysts of *Acanthamoeba* [62, 64, 67, 75, 81].

The use of both fluorescence microscopy and immunohistochemistry in brain and skin tissue sections is efficient methods to specifically detect *Acanthamoeba* [82–87]. Anti-*Acanthamoeba* antibodies generated in rabbits can be used to identify amoebae in tissue. Patient specimens are incubated with anti-*Acanthamoeba* antibodies followed by a secondary antirabbit IgG associated with a fluorescent marker to detect both trophozoites and cysts [74]. More recently, species-specific monoclonal antibodies were developed to use as an important diagnostic and epidemiological tool. These monoclonal antibodies recognize *A. castellanii, A. polyphaga, A. lenticulata*, and *A. culbertsoni*, react with formalin-fixed, paraffin-embedded infected tissues, and recognize both the trophozoite and cyst stages of the amoebae [88].

Transmission electron microscopy (TEM) also can be used as a tool to differentiate cysts and trophozoites of *Acanthamoeba* from host cells and from other amoebae, such as *B. mandrillaris* [63, 70, 89, 90]. However, this technique is expensive; sample preparation is time consuming and requires personnel with expertise.

#### 3.2.3. Culture Methods

Isolation and culture of *Acanthamoeba* can be performed by placing brain or skin biopsy/autopsy samples on 1.5% nonnutrient agar plates covered with a layer of *Escherichia coli or Enterobacter aerogenes* [3, 7, 48, 58, 83, 91]. Depending on the density of amoebae, *Acanthamoeba* can be observed after 24 hours of inoculation. Samples, also, can be placed on tissue culture cells in the presence of antibiotics (penicillin-streptomycin and gentamicin) in which case the amoebae destroy the cell monolayer in 24 to 48 hours depending on the number of amoebae present [3, 6, 59]. 

#### 3.2.4. Serology

To detect *Acanthamoeba* infections, an increase in antibody titer can be an indication of infection. For this evaluation, indirect immunofluorescence (IIF) is performed using serial dilutions of serum from an individual suspected of having Acanthamoebiasis, followed by incubation of the sera with slides containing fixed amoebae or amoebic extracts. The detection can be achieved by adding an anti-IgG antibody associated with a fluorescent label such as FITC, and antibody detection and titration can be determined by fluorescence microscopy [92]. *Acanthamoeba* infected individuals possess high antibody titers (between 1 : 256 and 1 : 1024) in serum while healthy individuals who have been exposed to *Acanthamoeba* in the environment have low antibody titers, usually not higher than 1 : 80 [82, 92–96]. Thus, IIF can be a useful tool to confirm infection in patients who are suspected of being infected with *Acanthamoeba* [96]. Western immunoblot analysis, also, has been used to demonstrate antibodies to *Acanthamoeba* in human serum [97]. An ELISA method utilizing whole fixed trophozoites rather than disrupted amoebic extracts as the antigen source was developed and shown to be an effective tool for identifying antibodies to *Acanthamoeba* in the clinical laboratory setting [98]. 

#### 3.2.5. PCR

Detection of *Acanthamoeba* can be rapidly achieved by using molecular methods. For diagnostic purposes, the detection of *Acanthamoeba* at the genus level is sufficient to recognize whether an individual is infected. Molecular identification of *Acanthamoeba* can be performed by polymerase chain reaction assays [99–101]. The complete DNA gene sequence of the 18S ribosomal RNA gene (18S rDNA) permitted the design of a reliable primer pair specific for *Acanthamoeba* genus, called JDP1 and JDP2. Use of the JDP1 (forward primer) 5′-GGCCCAGATCGTTTACCGTGAA and the JDP2 (reverse primer) 5′–TCTCACAAGCTGCTAGGGAGTCA, respectively, results in a specific amplimer of 500 bp, called ASA.S1. An advantage of this PCR assay is that it detects all known *Acanthamoeba* subgroups [100]. This PCR assay has been successfully used to detect *Acanthamoeba* in the environment as well as in patients with GAE and cutaneous acanthamoebiasis [83, 102–105]. However, DNA amplification from cysts is a troublesome task. Thus, treatment of cysts with proteinase K prior to DNA extraction has been suggested to increase the positive results by PCR assays [106]. 

Studies have shown that the majority of GAE and AK-causing amoebae have a specific PCR product (these amoebae are included in a subgroup called T4), when primers to amplify the small subunit rRNA genes (SSU rDNA) are used [107]. Mitochondrial DNA PCR, also, has been used successfully to detect *Acanthamoeba* from brain slices and CSF [108, 109]. Real-time PCR has been used as a fast tool to differentially identify free-living amoebae and to differentiate *Acanthamoeba* from *N. fowleri* and *B. mandrillaris* [57, 110]**. **A real time PCR assay developed by Rivière et al.,[110] utilizes Taqman technology to detect 18S ribosomal DNA (rDNA). This assay, based on the *Acanthamoeba* T4 genotype, does not detect other genotypes such as T10. The real time PCR assay developed by Qvarnstrom et al., [57] is a triplex assay to distinguish *Acanthamoeba* from other pathogenic FLA and is more complete in that it was designed to detect a broader range of *Acanthamoeba* genotypes. Both real time assays have been validated by testing a number of positive and negative clinical samples [111]. 

## 4. *Acanthamoeba spp.* and Amoebic Keratitis (AK)

In contrast to GAE, which is a chronic infection, AK is an acute, painful infection that can occur in immune competent individuals. This disease is related to the use of contact lenses or previous corneal trauma. When AK is not treated promptly, loss of visual acuity and blindness can occur [6, 7, 58, 59, 112–116]. 

### 4.1. Symptoms of AK

Initial symptoms of AK are not specific and include disproportional eye pain, photophobia, eye redness, and tearing, usually affecting one eye [116]. However, bilateral AK has been described, as a complication of the initial infection [116]. Using a slit-lamp, corneal inflammation leading to formation of a ring-like stromal infiltrate can be observed. Furthermore, corneal epithelial erosion, irregularities, and edema are present. The radial perineural distribution of the infiltrate (radial keratoneuritis) is characteristic for AK, similar to the type of infiltration observed in *Pseudomonas aeruginosa* keratitis [112–115, 117–120]. Later stages of infection can result in epithelial denudation and stromal necrosis. Contact lens usage and/or incidents of corneal trauma are strong indicators for AK [121–123]. Despite the clinical picture, AK is often misdiagnosed as herpes or bacterial keratitides which exhibit similar clinical symptomatology [114, 124, 125].

### 4.2. Diagnostic Methods

Diagnosis of AK can be undertaken by analysis of the clinical appearance of the cornea and by the demonstration of amoebae in the cornea [112, 125].

#### 4.2.1. Microscopic Methods

The detection of *Acanthamoeba* can be achieved by analysis of a corneal biopsy. However, corneal scraping has been an efficient and noninvasive method used to isolate amoebae and to diagnose *Acanthamoeba* keratitis [126]. After scraping, samples can be smeared on glass slides. Light microscopy is an efficient means to detect *Acanthamoeba* in corneal scrapings, in biopsy samples, and in keratoplasty specimens. *Acanthamoeba* can be detected in wet-mount preparations of corneal scraping, using 10% KOH [126–132]. Moreover, impression cytology was able to remove amoebic specimens of an AK patient [132]. H & E and Giemsa stains have been used successfully to detect both trophozoites and cysts of *Acanthamoeba*. Cysts stained by Giemsa or H & E are clear, bright with polyhedric or stellate shaped cysts, while trophozoites, with the central nucleolus and vacuoles, are more difficult to detect, since they can resemble inflammatory cells [112, 115, 129, 130, 133–135]. PAS stain also has been used for detection of cysts [128, 136]. As described previously, IIF assays and immunofluorescence microscopy can be used to detect *Acanthamoeba* in brain and skin tissue, as well as in corneal specimens, contact lenses, and lens cases [134, 137, 138]. Immunoperoxidase technique is also efficient to detect *Acanthamoeba* in host tissue [121, 134]. Cysts and trophozoites are easily detected by transmission electron microscopy [133]. 

Fluorescent stains have been used to detect *Acanthamoeba* cysts in corneal samples. Calcofluor white is a fluorescent compound that is able to bind cellulose in the cell wall of *Acanthamoeba* cysts found in corneal scrapings and paraffin-embedded sections of corneal tissue [138, 139]. Furthermore, specimens previously stained with H & E, and other stains can be stained subsequently with calcofluor white, which is rapid and efficient but requires a fluorescence microscope. It is also important to note that in mixed fungi, *Acanthamoeba* infections, both pathogens will be stained, since both amoebic cysts and fungi cell walls are possible targets for calcofluor white. Acridine orange, another fluorochromatic dye, has been used for rapid diagnosis of AK [78]. The use of fluorescent dyes may lead to false-positive staining patterns of cell debris [129, 130]; therefore, an experienced observer and a fluorescence microscope are necessary for a proper diagnosis.

The use of in vivo confocal microscopy to detect *Acanthamoeba* in corneal tissue at distinct depths, in real time, without any invasive procedure has been successfully used to diagnose AK [140–148]. In a recent Chicago-area outbreak, in conjunction with other methods, such as culturing or light microscopy, confocal microscopy was used to detect *Acanthamoeba* [148]. High-contrast rounded bodies, indicative of amoebic cysts, are commonly observed [143, 146, 147]. Limitations of confocal microscopy as a definitive diagnostic tool for AK have been presented [149]. However, studies have shown that when confocal microscopy was used by experienced operators, the technique is sensitive and specific for detection of *Acanthamoeba* in corneal tissue [148].

#### 4.2.2. Culture Methods

As described previously for GAE, culture of amoebae from corneal biopsies or scrapings and washes from contact lenses or lens cases is still the most common, cheap, and efficient method to detect *Acanthamoeba*. Samples from the infected cornea are inoculated on 1.5% nonnutrient agar plates covered with *E. coli* or other nonmucoid bacteria. The plates can be incubated at 28–30°C for days to weeks, which depends on the number of amoebae in the samples. The presence of amoebae can be checked by using an inverted microscope [48, 150]. 

### 4.3. PCR

As described previously for GAE and cutaneous lesions, PCR probes have been used, also, to confirm the presence of amoebae in corneal biopsies and scrapings, contact lenses, lens cases, lens solutions, and also in the environment [57, 100, 110, 151, 152]. Moreover, PCR is an efficient method to detect *Acanthamoeba* in tear samples [153], a completely noninvasive way to perform AK diagnostics. The sensitivity of PCR methods to diagnose AK was compared with direct microscopic examination and culture, and it was reported that PCR was more sensitive than morphological detection [154]. Two real time PCR assays have been validated to use as diagnostic tests for AK [57, 110]. However, it has been shown that a number of commonly used topical ophthalmic drugs have an inhibitory effect on PCR assays [155]. Thus, it is important that ophthalmologists rinse the eye surface extensively to remove any inhibitory substances to minimize the risk of misdiagnosis due to false negative PCR results. 

## 5. *Balamuthia mandrillaris * and *Balamuthia *Amoebic Encephalitis (BAE)

*B. mandrillaris* is the only species of *Balamuthia* known to cause infection in humans or animals. *Balamuthia* was first isolated from the brain of a mandrill baboon that died at the San Diego Zoo from meningoencephalitis. The amoebae was first described as a leptomyxid amoebae but later identified and named *Balamuthia mandrillairs* [156–159]. The life cycle of *Balamuthia* consists of a trophozoite (Figure 1(c)) and a cyst state. The amoeba is found in soil, but its presence in water has been suggested based on cases of BAE occurring in animals and humans that had a history of swimming in stagnant water [160–163]. Although *Balamuthia* is considered an opportunistic pathogen, it can cause disease in both immune compromised and immune competent individuals. Infections can occur in children and adults. The incubation period of BAE is extended, and therefore the source and mode of infection have not been definitively determined. It has been suggested that the portal of entry may be via cutaneous lesions, nasal passages, or inhalation via the respiratory tract with subsequent hematogenous spread to the brain and other organs [159]. 

### 5.1. Symptoms of BAE

The encephalitis caused by *B. mandrillaris*, also, is a rare disease with nonspecific symptoms. Symptoms of BAE are chronic and include headaches, nausea, vomiting, fever, myalgia, seizures, weight loss, hemiparesis, and speech difficulties, usually associated with previous skin granulomatous lesions. The aforementioned symptoms are confusing, since they are similar to other brain infections, including tuberculosis, toxoplasmosis, cysticercosis, meningitis, and also brain tumors [7, 158, 164–169]. 

### 5.2. Diagnostic Methods

#### 5.2.1. Imaging Methods

Brain lesions caused by *B. mandrillaris* can be detected by neuroimaging, such as CT scans and MRI [170–172]. Focal enhancing lesions, cystic lesions, edema, and hydrocephalus can be observed [172, 173]. The lesions can mimic other types of disease, such as gliomas, brain abscesses, and hematomas [7]. Thus, the lack of specificity makes the proper diagnosis by imaging methods difficult.

#### 5.2.2. Microscopic Methods

Light microscopy can be used to detect the presence of *B. mandrillaris* in host tissue. *Balamuthia* has often been identified as *Acanthamoeba* in tissue because both amoebae cause granulomatous amoebic encephalitis. Amoebae can be observed from brain and skin biopsies and autopsies [174–176]. Unlike infection with *N. fowleri*, *B. mandrillaris* is generally not seen in CSF preparations, although it has been isolated from CSF of a 33-year-old patient that died of BAE [7, 177]. In the majority of cases described, *B. mandrillaris* was observed in brain biopsy specimens embedded in paraffin and processed for H&E [158, 170, 175, 178]. Areas of inflammation, granulomas, and the presence of trophozoites and cysts of *B. mandrillaris*, especially around blood vessels, are observed [165, 169, 179]. Trophozoites show an oval-to-round shape, with a single nucleus and a large nucleolus, while cysts are rounded with a thick wall. However, these morphological characteristics are not adequate to differentiate *B. mandrillaris* from *Acanthamoeba spp.*, and also the ability to differentiate these amoebae from host macrophages requires expertise [7, 84].

Antibodies are crucial to the specific detection of *B. mandrillaris* in tissues. Studies have confirmed, both in biopsies and autopsies, the presence of *B. mandrillaris* in brain and skin tissue [84, 157, 170, 174, 178]. Usually, paraffin-embedded specimens are sectioned and incubated with rabbit anti-*Balamuthia* serum, and a FITC-conjugated secondary antibody against rabbit IgG is used to detect amoebae with high degree of specificity [159].

It is possible to identify *B. mandrillaris* in biopsies with the use of transmission electron microscopy (TEM). Unlike host cells, trophozoites contain a characteristic dense nucleolus, and the cytoplasm contains many vesicles. Furthermore, TEM can distinguish *B. mandrillaris* from *Acanthamoeba*, since *B. mandrillaris* possesses a tripled-walled cyst, a distinctive trait when compared to the doubled-walled cyst of *Acanthamoeba* [158, 159].

#### 5.2.3. Culture Methods

Isolation of *B. mandrillaris* from biopsy specimens is possible; however amoebic growth is slow and also requires the use of tissue culture cells as a food source, since *B. mandrillaris* does not feed on bacteria [48, 157–159]. This is not a recommended method.

#### 5.2.4. Serology

One of the characteristics of *B. mandrillaris* infection is the high concentration of antibodies to the amoebae in host serum [180–182]. It is possible to determine an infection in humans suspected of having BAE by the presence of antibodies against *B. mandrillaris* in their sera using enzyme-linked immunoassays (ELISAs). This methodology was used to screen a group of encephalitis patients in California, and it was shown that 7 individual samples from 290 were positive for *B. mandrillaris* [181]. Antibodies to *B. mandrillaris* do not cross-react with other amoebae [180]. Thus, ELISA technique can be useful for screening of samples containing large number of individuals. More recently, flow cytometry was successfully used to detect and quantify antibodies against *B. mandrillaris* in both healthy and diseased individuals [182].

#### 5.2.5. PCR

Rapid and highly specific *B. mandrillaris* detection can be achieved by using polymerase chain reaction methodology. *B. mandrillaris* is a well-defined phylogenetic species, with no SSU rDNA sequence variation between isolates and low levels of mitochondrial DNA variation. Booton and coworkers [183, 184] developed specific primers from a portion of the mitochondrial rRNA gene (rns). The PCR reaction of these primers resulted in a 1075 bp product, where the product is specific for *B. mandrillaris* and not for *Acanthamoeba*. Detection of *B. mandrillaris* was performed successfully on clinical samples using these primers, both in brain tissue, and CSF [177, 185, 186]. The sensitivity of the PCR detection was tested, and it was observed that 0.2 amoebae were enough for amplification, since mitochondrial DNA had been used [186], indicating that the employment of PCR was an efficient diagnostic tool. The efficacy of PCR method was compared with IIF as a diagnostic tool on archival brain tissue and a high degree of agreement was reported [109]. By using distinct primers, a multiplex real time PCR to detect *B. mandrillaris* was developed, with rapid test completion time and high sensitivity, detecting one amoebae per sample [57]. This multiplex assay has been validated and is recommended for detection of FLA in clinical samples [111]. More recently, a real time PCR assay was developed for *B. mandrillaris*, targeting the RNAase P gene [187]. Sensitivity and specificity were observed, with the probes able to detect small amounts of amoebic DNA.

## 6. *Sappinia pedata * and *Sappinia *Amoebic Encephalitis (SAE)

The free-living amoeba*, Sappinia*, a newly discovered human pathogen of the central nervous system (CNS), can cause amoebic encephalitis in humans [9, 10]. There are two species of *Sappinia*, *S. pedata* and *S. diploidea*. *Sappinia* has a worldwide distribution and has been isolated from elk and buffalo feces, soil contaminated with bovine feces, decaying ground plant litter and fresh water [188, 189]. The life cycle of *Sappinia* consists of two stages, a trophozoite and a cyst [190]. The first and only case of amoebic encephalitis caused by *Sappinia sp.* occurred in a previously healthy immune competent adult male who survived the infection. The incubation period and the route of infection are unknown but thought to be by inhalation through the nasopharynx or by hematogenous spread to the brain [9]. The amoeba that caused this encephalitis was originally identified as *S. diploidea* but has now been identified as *S. pedata* using molecular techniques to identify the amoeba (191,192). It is not known whether the other species (*S. diploidea*) can cause infections in human or animal hosts. 

### 6.1. Symptoms of SAE

In the single case of *Sappinia* amoebic encephalitis that has been reported, a sinus infection occurred prior to the onset of symptoms. The individual developed nausea, vomiting, bifrontal headache, photophobia, and blurry vision. A loss of consciousness occurred for a brief period [7, 9, 10, 191]. A successful outcome in this patient was reported after surgical excision of a tumor-like mass in the brain and treatment using azithromycin, intravenous pentamidine, itraconazole, and flucytosine [10]. 

### 6.2. Diagnosis

A solitary tumor-like mass without an abscess wall in the brain can be observed by MRI for *Sappinia* encephalitis. The excised mass or biopsy tissue can be fixed and embedded in paraffin. Brain sections stained with H & E may exhibit necrotizing hemorrhagic inflammation containing amoebae. Eosinophils and granuloma formation are lacking. *Sappinia* amoebae can be distinguished from other FLA by the presence of a distinctive double nucleus in which the 2 nuclei are closely apposed with a central flattening [188, 189]. Two nucleoli are found in the double nucleus. These structures can be observed in paraffin sections stained with H & E, Giemsa, or Periodic Acid Schiff. The amoebae are readily observed in cryostat sections stained with H & E [7, 10, 191]. Brain tissue can be fixed in glutaraldehyde and prepared for transmission electron microscopy to visualize the amoebae in tissue. 

### 6.3. PCR

*Sappinia pedata* and *S. diploidea* can be identified and distinguished by amplification of the SSU rDNA using universal eukaryotic SSU primers followed by an Internal Transcribed Spacer PCR assay. Primers used in the ITS PCR assay are ITS1–P1F5′-GTA ACA AGG TATCCG TAG GTG AAC C-3′ and ITS2–P4R: 5′TCC TCC GCT TAT TGA TAT GC—3′ [190]. The amoebae originally identified as *S. diploidea* in the single reported case of *Sappinia* amoebic encephalitis was later identified as *S. pedata* using newly developed real-time PCR assays based on 18S rRNA gene sequences [191]. The assay specific for *Sappinia* can be incorporated into the multiplex PCR assay described by Qvarnstrom et al., [57] that distinguish *Acanthamoeba, B. mandrillaris*, and *N. fowleri* for simultaneous detection of the four genera of FLA that cause infections in humans [191]. 

### 6.4. Culture

*Sappinia* can be cultured on nonnutrient agar coated with bacteria and on tissue culture cells [7].

## 7. Conclusion: The Importance of Early Diagnosis

Although infections with FLA are considered rare, there has been an increase in the number of reported cases in recent years. CNS infections caused by pathogenic free-living amoebae (FLA) are for the most part fatal. Corneal infection caused by *Acanthamoeba* can lead to blindness or vision impairment in AK. Recently, it has been shown that there is a greater chance of cure if these infections are detected early and treated timely. However, a fast and efficient diagnosis depends on two variables: the familiarity of practitioner with the symptomatology and treatment of FLA infections, and also the appropriate material to process for a fast and definitive diagnosis. At the present time, it is not known whether FLA infections are rare because they are underreported or due to misdiagnosis. Most infections have been diagnosed postmortem. It is not possible to determine the rarity of these infections, since these infections are relatively unknown, and in many cases autopsies are not performed.

The number of contact lenses users around the world has increased, and consequently AK outbreaks have been more frequent, which requires a faster public health response. GAE and cutaneous acanthamoebiasis can be one of the most frequent secondary diseases in AIDS-HIV patients and in other immunosupressed individuals, since *Acanthamoeba* is found throughout the world. The dramatic increase in BAE could reflect the potential of this infection to be a common worldwide disease. The increased incidence of PAM caused by *N. fowleri* in recent years may be due to greater recreational activity in warm water lakes and parks. PAM could be avoided with greater awareness of the disease, using public education programs, closing of swimming pools that are improperly chlorinated, and wearing nose clips while diving and engaging in water activities when the nose is submerged. The recent finding of *S. pedata* as a pathogen of the CNS in humans suggests that other free-living amoebae, not yet identified, may be causative of amoebic of amoebic encephalitis. 

A more rapid clinical response could provide higher survival rates since treatment is available. Thus, fast and efficient diagnostic tests are pivotal for treatment success. The availability of PCR probes is a promising procedure to obtain fast and specific, confirmatory diagnosis of PAM, GAE, AK, BAE, and SAE in a timely fashion for efficient treatment. To this end, a multiplex PCR assay is available and has been validated as an important and specific tool to identify, *N. fowleri, Acanthamoeba spp.,* and *B. mandrillaris* in clinical specimens [57, 111] but only a few reference laboratories are capable of doing diagnostic detection of FLA [191]. Additionally, a PCR assay has been developed to detect *Sappinia* in clinical samples [191]. In conclusion, the recognition of these diseases and specific diagnostic tests could lead to earlier treatment and diminish the severity and lethality of these infections in the human host.
